# The Tariff on Surgical Instruments

**Published:** 1888-03

**Authors:** J. C. LeHardy

**Affiliations:** Corresponding Secretary Georgia Medical Society; Savannah, Ga.


### THE TARIFF ON SURGICAL INSTRUMENTS.

---

*To the Editors of Atlanta Medical and Surgical Journal:*

At the annual meeting of the Georgia Medical Society, held January 3d, 1888, the following resolution was unanimously carried:

*Resolved*, That the Corresponding Secretary enter into correspondence with the medical journals of the country in order to enlist their influence in support of the movement to remove the import duties from all medical and surgical instruments and appliances, including those used in the diagnosis as well as treatment of disease, so that they may be furnished to those needing them at the lowest possible price.

In compliance with the above resolution, I wish to solicit your earnest attention and a notice in your publication, which will claim the attention of your readers, hoping that your country readers, especially, will appreciate the truth and importance of our proceedings.

Perhaps the statement of a few facts will assist the reader in realizing the extent of the grievance and the justice of the plea for which we ask co-operation.

1st. Physicians are at the mercy of instrument-makers in regard to price, make and quality of finish because of the lack of sufficient competition.

2d. The price of instruments made in this country is out of proportion to that paid for similar instruments on the continent of Europe.

3d. Surgical instruments and appliances are so costly that but few doctors entering the profession can provide themselves with an outfit adequate to carry on a general practice. At present prices it is impossible for a country physician's income to sustain his investing in costly instruments, and as a result many simple cases, such as retention of urine, foreign bodies in nose or throat, deep-seated abscesses, etc., all of which could be relieved at once with the proper instruments, must either die from the immediate cause or from the effects of time lost in seeking skillful manipulation, or else they are frequently crippled and disfigured because the most intelligent help, though patiently given, is itself crippled for want of proper instruments.

4th. The cheaper grades of instruments are either antiquated or so poorly made that they may prove a cause of failure in operations, sapping, as it were, the natural inclinations to surgery in its inception.

5th. European instruments are from 25 to 75 per cent. cheaper than ours, and their introduction into the market will enable the mass of doctors to buy those of prime necessity, will bring down the price of home-made appliances and oblige the makers to use good material and put a better finish to their work.

6th. The removal of import duties on surgical and other instruments used by the profession, and on medicines in general, will produce the same results, as we all know it did on the article of quinine. Respectfully, J. C. LEHARDY,

*Corresponding Secretary Georgia Medical Society.*

Savannah, Ga., January, 1888.

